# Leveraging the Attributes of *Mucor hiemalis*-Derived Silver Nanoparticles for a Synergistic Broad-Spectrum Antimicrobial Platform

**DOI:** 10.3389/fmicb.2016.01984

**Published:** 2016-12-15

**Authors:** Nafe Aziz, Rishikesh Pandey, Ishan Barman, Ram Prasad

**Affiliations:** ^1^Amity Institute of Microbial Technology, Amity University, NoidaIndia; ^2^Connecticut Children’s Innovation Center, FarmingtonCT, USA; ^3^Department of Pediatrics, University of Connecticut Health, FarmingtonCT, USA; ^4^Department of Mechanical Engineering, Johns Hopkins University, BaltimoreMD, USA; ^5^Department of Oncology, Johns Hopkins University, BaltimoreMD, USA

**Keywords:** silver nanoparticles, *Mucor hiemalis*, antibacterial activity, antifungal activity, green synthesis

## Abstract

Driven by the need to engineer robust surface coatings for medical devices to prevent infection and sepsis, incorporation of nanoparticles has surfaced as a promising avenue to enhance non-fouling efficacy. Microbial synthesis of such nanoscale metallic structures is of substantive interest as this can offer an eco-friendly, cost-effective, and sustainable route for further development. Here we present a *Mucor hiemalis*-derived fungal route for synthesis of silver nanoparticles, which display significant antimicrobial properties when tested against six pathological bacterial strains (*Klebsiella pneumoniae, Pseudomonas brassicacearum, Aeromonas hydrophila, Escherichia coli, Bacillus cereus*, and *Staphylococcus aureus*) and three pathological fungal strains (*Candida albicans, Fusarium oxysporum*, and *Aspergillus flavus*). These antimicrobial attributes were comparable to those of established antibiotics (streptomycin, tetracycline, kanamycin, and rifampicin) and fungicides (amphotericin B, fluconazole, and ketoconazole), respectively. Importantly, these nanoparticles show significant synergistic characteristics when combined with the antibiotics and fungicides to offer substantially greater resistance to microbial growth. The blend of antibacterial and antifungal properties, coupled with their intrinsic “green” and facile synthesis, makes these biogenic nanoparticles particularly attractive for future applications in nanomedicine ranging from topical ointments and bandages for wound healing to coated stents.

## Introduction

Microbial infections have presented a persistent threat to human health with infections emanating in traumatic and surgical wounds and burns posing an overarching challenge, despite the pioneering breakthroughs in antibiotics and antiseptics (Murray, 2007). Excessive antibiotic use (and misuse) has resulted in growing resistance to such treatment strategies (MacGowan, 2001; Hancock, 2007). Notably, the outbreak of antibiotic-resistance has resulted in an unprecedented rise in nosocomial infections, such as urinary tract infections, pneumonia, and bloodstream infections. In the United States alone, roughly 1.7 million estimated nosocomial infections, primarily from bacteria and fungi, are responsible for 99,000 deaths each year (Klevens et al., 2007). Owing to this increased resistance of several pathogenic bacteria against antibiotics, considerable attention has been focused on developing new treatment modalities featuring alternative antibacterial agents (Aziz et al., 2014; Manikprabhu and Lingappa, 2014; Teillant et al., 2015). In this milieu, metallic silver, especially in the form of nanoparticle and nanostructured substrates, has received considerable traction for antibacterial activity due to its unique physiochemical properties (Husain et al., 2015; Prasad et al., 2016). While the antimicrobial activity of silver has been known for several centuries (Demling and De Santi, 2001), recent developments in the field of nanocrystalline silver have led to renewed interest (Paddock et al., 2007).

Recent reports in the literature have illustrated the bactericidal activity of silver nanoparticles (AgNPs; Rai et al., 2009; Aziz et al., 2015). Ingle et al. (2008) have shown that AgNPs exhibited significant antimicrobial activity against *Escherichia coli* and multidrug resistant *Staphylococcus aureus*, while Pal et al. (2007) have studied the impact of nanoparticle architecture on the antibacterial activity against *E. coli*. Importantly, AgNPs is also found to disrupt the formation of biofilm (Husain et al., 2015), which exhibits an intricate structure composed of bacterial cells embedded in an matrix of extracellular polymeric substances and serves as an effective barrier against antibiotics and the host immune system. Bacteria residing in biofilms are observed to be significantly more resistant to antibacterial compounds with respect to their planktonic form and therefore the ability of AgNPs to interfere with bacterial biofilms affords a key opportunity for biomedical applications. The most notable application of AgNPs in the medical industry include topical ointments (Tian et al., 2007; Hebeisha et al., 2014), while newly devised AgNPs-coated dressings also appear promising for the management of wounds and infections (Maneerung et al., 2008; Wilkinson et al., 2011; Nam et al., 2015).

To meet the synthesis demands for topical ointments and wound dressings, numerous physical and chemical routes are commonly used to produce a palette of controlled and tailored Ag nanostructures (Xie et al., 2007). However, the chemical reagents used normally for nanoparticles’ synthesis and stabilization are toxic and lead to non-ecofriendly by-products (Ingale and Chaudhari, 2013; Tripathi et al., 2016). Alternately, investigators have also proposed the use of microbial platforms as a “green” and sustainable substitute for nanoparticle synthesis (Nel et al., 2009; Siddiqi and Husen, 2016). Microbes have a promising role in the fabrication of nanoparticles due to their natural mechanism for detoxification of metal ions through reduction that can be achieved in an extra- or intracellular manner. Unlike nanoparticles derived from physical and chemical methods, biogenic nanoparticles have intrinsic protein capping and stabilizing agent on the surface that confers rare physiological solubility and stability (Ahmad et al., 2003). In addition to being eco-friendly, the emerging microbial synthesis route is also economical owing to significant reduction in downstream processing requirements. Inspired by the microbe’s ability to function under divergent extremes of temperature, pressure, and pH, several bacterial and algal chassis have been explored over the last decade by several groups, including our own laboratory (Nanda and Saravanan, 2009; Jain et al., 2011; Pantidos and Horsfall, 2014; Aziz et al., 2015; Patel et al., 2015). Notably, fungal-derived nanoparticle production shares these salient advantages and further combines facile scale up opportunities, economic viability, convenient processing and biomass handling, and faster biosynthesis rate in cell-free filtrate (due to the higher amount of proteins secreted in fungi with respect to bacteria) (Prasad et al., 2016). Moreover, the presence of fungal mycelia provides an increased surface area with the nanoparticles precipitated outside the cell being devoid of unnecessary cellular components and therefore amenable to direct use in applications of interest (Varshney et al., 2012; Kitching et al., 2014). However, there are few systematic studies that examine the potential synergism of fungal-derived AgNP with antibiotics/fungicides across a diverse spectrum of pathogenic microbial species.

In the present study, we demonstrate the antimicrobial efficacy of AgNP derived from such a fungal chassis featuring a representative member of the Mucorales species namely *Mucor hiemalis*. To the best of our knowledge, this is the first report employing the commonly occurring pathogenic fungus *M. hiemalis* for AgNP synthesis, which is particularly intriguing as this fungal species exhibits high growth rate and is easy to harvest with less cultivation time. Our X-ray diffraction (XRD) studies in conjugation with transmission electron microscopy (TEM) reveal the crystalline nature and size reproducibility of these biogenic nanoparticles. Furthermore, to decipher the molecular structural information of the functional moieties in the biosynthesized AgNP, Fourier transform infrared spectroscopy (FTIR) experiments were carried out. Importantly, we observe that these biogenic nanoparticles display competitive antimicrobial activity (in relation to established antibiotics) when incubated with different bacterial (Gram-positive and Gram-negative) and fungal pathogens and also show substantial synergistic effect when combined with known antimicrobial agents *in vitro*. Collectively, our findings offer an alternate fungal platform for production of silver nanoparticle and pave the way for animal model studies to examine the synergistic effect of the fungal-derived nanoparticle and antibiotics in retarding and preventing microbial infections *in vivo*.

## Materials and Methods

### Chemicals and Microbes

All chemicals used in this study were of analytical grade, procured from Sigma-Aldrich or Merck, India and used as received. All culture media were purchased from HiMedia, India. Double distilled water (ddH_2_O) or Milli-Q water was used for all the experiments in this study. For the antimicrobial efficacy study, six bacterial strains namely *Klebsiella pneumoniae* (KJ938546), *Pseudomonas brassicacearum* (KJ938545), *Aeromonas hydrophila* (KM104684), *Escherichia coli* (MCC 2412), *Bacillus cereus* (MCC 2243), and *Staphylococcus aureus* (MCC 2408) were used. The first three strains were procured from the Amity University (Noida, India), while the latter three along with the fungal strain *Candida albicans* (MCC 1151) were received from the Microbial Culture Collection at National Centre for Cell Science, Pune, India. Additionally, *Fusarium oxysporum* (NFCCI 245), *Aspergillus flavus* (NFCCI 384), and *Mucor hiemalis* (NFCCI 2228) were procured from National Fungal Culture Collection of India, Pune, India.

### Synthesis of AgNPs

*Mucor hiemalis* was grown on potato dextrose broth. Briefly, the pH of the medium was adjusted to 5.1 ± 0.2 and flasks were incubated at 180 rpm at 25°C for 72 h. The pure culture of *M. hiemalis* was maintained on potato dextrose agar (PDA) slants at 4°C. The mycelia were separated by centrifugation (5000 rpm) for 10 min and filtered using Whatman filter paper No. 1, and washed thrice with deionized water. After washing, 100 ml of deionized water was added to 10 g of wet fungal biomass and incubated for 48 h (180 rpm and 25°C). After incubation, the mycelia were again centrifuged (5000 rpm, 10 min) and filtered. Twenty milliliters of resulting aqueous extract was diluted with 80 ml of water followed by addition of 0.017 g (1 mM) silver nitrate (AgNO_3_) in a conical flask and stirred at 25°C in dark condition. Control experiment without AgNO_3_ was also carried out under identical conditions. After 24 h, visual observation revealed a color change—from colorless to reddish brown due to reduction of AgNO_3_ to AgNPs—of the fungal filtrate. Prior to characterization, 100 ml of the nanoparticle solution was centrifuged at 5000 rpm for 10 min. The supernatant was again centrifuged at 10,000 rpm for 60 min to obtain a pellet. The pellet was re-dispersed into 1 ml of deionized water (Kayalvizhi et al., 2014). Centrifugation and re-dispersion in ddH_2_O were repeatedly carried out to ensure better elimination of these extraneous entities followed by lyophilization to obtain powdered nanoparticles. The AgNP yield was determined by weighing the powdered nanoparticles.

### Nanoparticle Characterization

To examine the bioreduction of AgNO_3_ to AgNPs the absorption spectra were recorded at different temporal points using a double beam UV-Vis spectrophotometer (UV-1800, Shimadzu, Japan). The surface morphology of the biogenic AgNPs was characterized using scanning electron microscope (SEM; Zeiss Evo HD, Jena, Germany) and energy dispersive X-ray analysis (EDX, Oxford Instruments, UK) connected with the SEM. Experiments with acquisition time ranging from 60 to 100 s and an accelerating voltage of 20 kV was performed to confirm the presence of elemental silver inside the biologically synthesized nanoparticles. TEM (Philips, EM-410LS, JEOL, Japan) was also used to observe the morphology of the biosynthesized AgNPs. Acquired images were analyzed using Image J (http://imagej.nih.gov/ij/download.html). For microscopy observations, the sample was prepared by dispersing 5 mg of nanoparticles in 1 ml ethanol and the resulting mixture was sonicated for 30 min. A small drop of the resulting suspension was evenly spread on the grid followed by drying at room temperature and the samples were gold-coated before imaging. Additionally, for vibrational characterization, FTIR measurements (Varian 7000 FTIR, USA) were performed on the KBr pellet prepared with diluted AgNPs. Spectroscopic measurements were carried out in 400–4000 cm^-1^ range at a resolution of 4 cm^-1^. To evaluate the crystallinity of AgNPs, XRD (Ultima IV, Rigaku, Japan) measurements were also performed and the data was recorded at 2𝜃 range of 20° to 80° having K-beta filter with X-Ray of 1.54056 Å at 40 kV and 30 mA.

### Antimicrobial Properties

Antibacterial and antifungal activities of AgNPs were evaluated using agar well diffusion and disc diffusion method. To determine the antibacterial activities of AgNPs, culture of four Gram-negative bacteria namely *Klebsiella pneumoniae* (Kp), *Pseudomonas brassicacearum* (Pb), *Aeromonas hydrophila* (Ah), *Escherichia coli* (Ec) and two Gram-positive bacteria, namely *Bacillus cereus* (Bc) and *Staphylococcus aureus* (Sa), were prepared by growing a single colony overnight in nutrient broth and adjusting the turbidity to 0.5 McFarland standards. Hundred microliters of bacterial test pathogens were spread onto 25 ml Mueller-Hinton agar medium in the plates. Fifty microliters of different concentrations (5, 10, 20, and 30 μg/ml) of AgNPs and 50 μl of 10 μg/ml antibiotics (streptomycin, tetracycline, kanamycin, and rifampicin) were poured in the wells. Synergistic effect of AgNPs with respective antibiotics was evaluated at 10 μg/ml concentration both for AgNPs and antibiotic. These plates were incubated at 37°C for 24 h and the zones of inhibition were measured. All experiments were simultaneously performed in triplicates.

The antifungal activity was assessed using agar well diffusion method for *Candida albicans* while the disc diffusion method was used for *Fusarium oxysporum*, and *Aspergillus flavus*. The fungal spores were washed with sterile 0.85% saline containing 0.1% tween 80 (v/v). The spore suspension was adjusted with sterile saline to a concentration of 1.0 × 10^7^ spores/ml in a final volume of 100 μl per well. The inocula were stored at 4°C for further use. Dilution of the inocula was cultured on solid PDA to verify the absence of contamination and to check the validity of the inoculum. 15, 30, 45, and 60 μg/ml concentrations of AgNPs and 30 μg/ml of standard commercial fungicides [amphotericin B (A), fluconazole (F), and ketoconazole (K)] were poured into Petri plates and their growth inhibition was observed. Synergistic effect of AgNPs with respective fungicides was measured at 30 μg/ml concentration of the former. All experiments were simultaneously performed in triplicates.

### Antimicrobial Interaction Analysis

In order to assess the nature of the *in vitro* interactions between any nanoparticle–drug combinations, the data obtained from the zone of inhibition studies were quantified using the following equation:

(1) $$\begin{array}{c} I=\frac{I_{\left(A+B\right)}}{I_{A}+I_{B}} \end{array}$$

Where *I* is the estimated interaction index, *I*_(_*_A_*_+_*_B_*_)_ is zone of inhibition for the combination of AgNP and antibiotic/fungicide, *I_A_* is zone of inhibition of AgNPs alone and *I_B_* is zone of inhibition for antibiotic/fungicide. While the interactions are concentration dependent, since the concentrations of each of these agents was kept constant (equal to 10 μg/ml and 30 μg/ml for the antibacterial and antifungal interaction studies, respectively) the estimated interaction index in Eq. 1 is concentration independent. Importantly, if the 95% confidence intervals of *I* values of a combination of agents were significantly higher or lower than 1, synergy or antagonism, respectively, was concluded to be present for that particular combination.

### Statistical Analysis

Data analysis was carried out using standard statistical software GraphPad Prism. Descriptive statistical measures, especially the mean and standard deviation, were used to summarize the collection of data for each measurement. Two-way analysis of variance was used to evaluate the influence of independent variables as well as possible interactions between them in the antimicrobial efficacy study. Tukey’s procedure was used to determine whether the data show evidence of difference between the various classes of antimicrobial agents.

## Results

### Fungal-Derived AgNPs: Synthesis and Characterization

**FIGURE 1:**
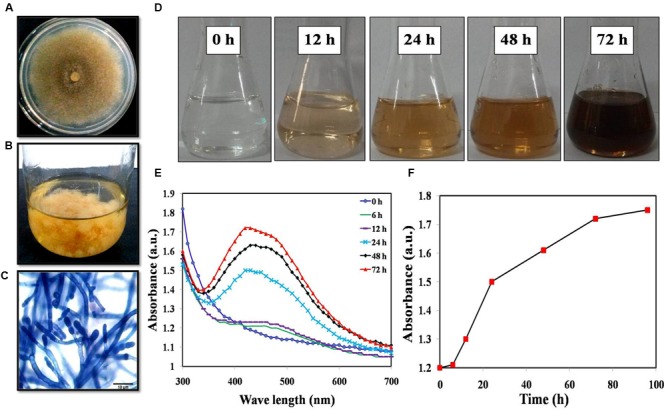
**(A)** Colony morphology; **(B)** Mass culture of *Mucor hiemalis*; **(C)** Microscopic view of *M. hiemalis* processed with lactophenol cotton blue stain; **(D)** Optical images of biosynthesized AgNP showing a range of colors from colorless (start of biosynthesis) to reddish-brown after 24 h; **(E)** UV-Vis spectra of *M. hiemalis*-derived AgNPs at various reaction times; **(F)** Reaction saturation curve indicating the evolution of the absorption band as a function of time.

*Mucor hiemalis* cultures were first grown in the potato dextrose medium for 3 days (**Figures 1A,B**). **Figure 1C** displays the representative microscopic morphology of the culture following staining with lactophenol cotton blue stain. Incubation of the cell extracts with 1 mM AgNO_3_ solution resulted in the formation of AgNPs and we observed a steady color change of the solution (**Figure 1D**). The formation of AgNPs was confirmed using UV-Vis absorption measurements at different time intervals (**Figure 1E**) with the spectrum obtained at 72 h displaying an absorbance maximum at 420 nm. As seen in **Figure 1F**, the peak intensity increases over time before plateauing around 72 h. Here, we obtained ca. 45 mg/100 ml of AgNPs from the fungal biosynthesis process and the quantity was consistent across three repeats.

**FIGURE 2:**
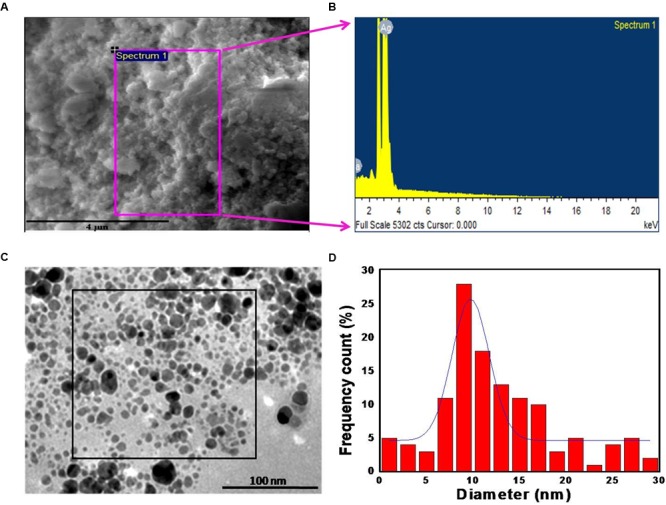
**Morphological and chemical characterization of *M. hiemalis*-derived AgNP. (A)** SEM image of biogenic AgNPs (scale bar indicates 4 μm); **(B)** EDX spectrum of biogenic AgNPs; **(C)** TEM image of the biogenic AgNPs (scale bar indicates 100 nm); **(D)** histogram of the size distribution of biogenic AgNPs.

**Figure 2A** shows a representative SEM image of the biologically synthesized AgNPs. On the region of interest (highlighted by the magenta square in the figure), we performed EDX measurement to determine the elemental composition of the synthesized nanoparticles (**Figure 2B**). The measured composition was consistent with the expected makeup with Ag accounting for more than 90% of the compositional contributors in the probed region (Supplementary Table S1). The remaining content of carbon, oxygen, and sodium reflect the presence of microbial biomolecules. To further investigate the size distribution, we performed TEM analysis on the *M. hiemalis*-derived AgNPs (**Figure 2C**). TEM images depict the spherical morphology of the nanoparticles and the particle size distribution was observed to vary in the range of 5–15 nm with only a very small fraction of the particles having dimensions between 20 and 30 nm (**Figure 2D**).

**FIGURE 3:**
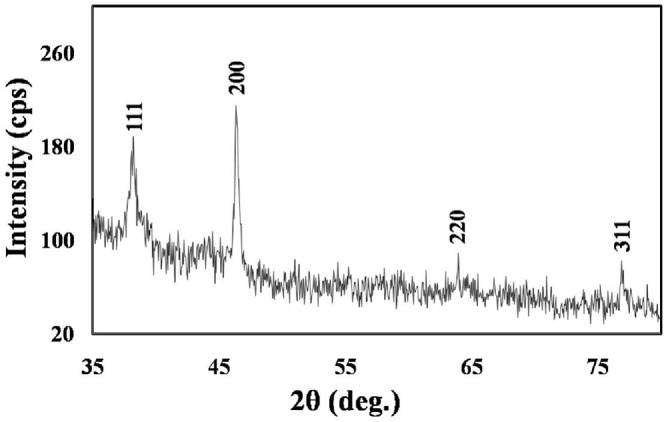
**XRD spectrum showing the crystalline nature of the *M. hiemalis*-derived AgNPs.** Further characterization details are included in Section “Results.”

As illustrated in the XRD graph of the biologically synthesized AgNPs (**Figure 3**), four peaks at 2𝜃 values of 38.3°, 46.4°, 63.9°, and 77.0° were observed corresponding to (111), (200), (220), and (311) planes, respectively, of silver that were in agreement with the standard powder diffraction card of Joint Committee on Powder Diffraction, silver file No. 04-0783 (Yang et al., 2011). The XRD results, thus, indicate that the synthesized nanoparticles have a distinctly crystalline nature. We computed the mean size of the ordered (crystalline) domains (D) using the Debye–Scherrer formula:

$$\begin{array}{c} D=0.9\;\lambda /\beta \cos\;\theta \end{array}$$

where λ represents the X-ray wavelength (0.1541 nm), β is the line broadening at half the maximum intensity (equivalent to the full width at half maximum (FWHM)) and 𝜃 is the Bragg’s angle. Based on the different 2𝜃 values, one can estimate a range of average crystalline sizes that is consistent with the previous TEM measurements of **Figure 2D**.

**FIGURE 4:**
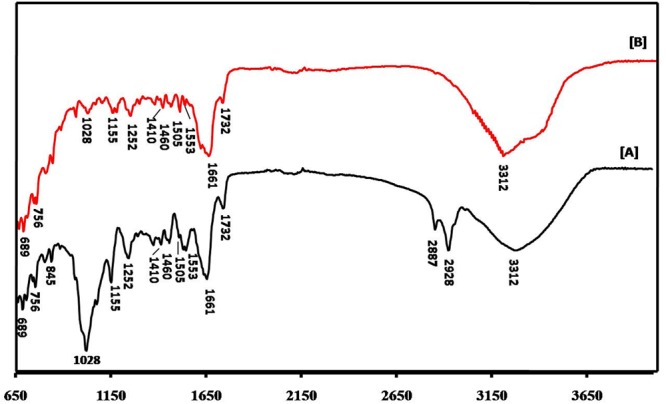
**FTIR spectra of (A)** cell extract and **(B)** *M. hiemalis*-derived AgNPs.

The FTIR spectra of the cell extract and the biologically synthesized AgNPs are presented in **Figure 4**. The notable peaks at 3312, 1661, and 1028 cm^-1^ in the FTIR spectrum of the extract can be ascribed to the O–H stretching vibration of aromatic compounds (such as phenols), C=C stretching vibration of alkenes and C–N stretching, respectively. The features at 2928 and 2887 cm^-1^ can be ascribed C–H stretching vibrations, whereas 1732 and 1553 cm^-1^ is likely indicative of C=O stretching vibration of ketones and NH_2_ bending vibration of the primary amines, respectively. Furthermore, the bands at 1460, 1252, and 1155 cm^-1^ are consistent with peaks representative of CH_2_ group bending, asymmetric PO_2_^-^ stretching mode, and C–O stretching mode of the C–OH groups of amino acids. Notably, a gross inspection of **Figures 4A,B** reveals that the FTIR spectrum of the AgNP solution is considerably less intense and is broadened than that acquired from the cell extract. For instance, the spectral bands at 2928 and 2887 cm^-1^ of the cell extract is barely perceptible in the spectrum of the synthesized AgNPs. Notably, reduction in the intensity of the 1028 cm^-1^ peak after biosynthesis of AgNPs suggests that one or more cyclic peptides is involved in synthesizing and capping the nanoparticles.

### Antimicrobial Activity

**FIGURE 5:**
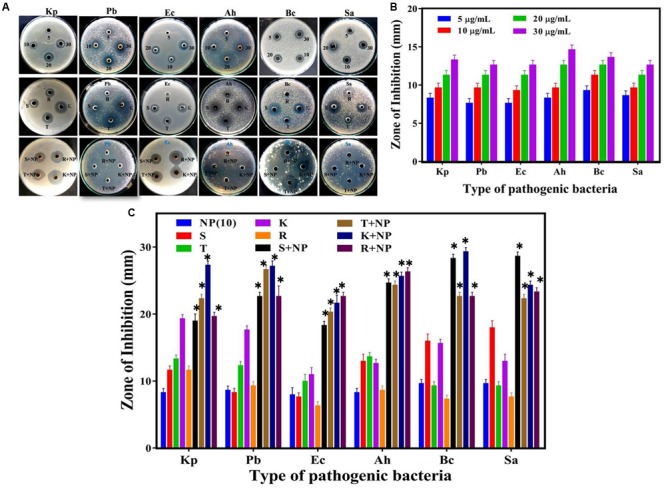
**Comparative analysis of antibacterial efficacy of *M. hiemalis*-derived AgNP. (A)** Zone of inhibition against four Gram-negative bacterial pathogens, *Klebsiella pneumoniae* (Kp), *Pseudomonas brassicacearum* (Pb), *Escherichia coli* (Ec), and *Aeromonas hydrophila* (Ah), and two Gram-positive bacterial pathogens, *Bacillus cereus* (Bc) and *Staphylococcus aureus* (Sa); **(B)** Measurements of the antibacterial activity of AgNPs (NP) at 5, 10, 20, and 30 μg/ml concentration against the six bacterial pathogens; and **(C)** Effect of biologically synthesized AgNPs (NP) and antibiotics [streptomycin (S), tetracycline (T), kanamycin (K), and rifampicin (R)] used individually against the bacteria and in combination. The potential synergistic effect of biologically synthesized AgNPs with antibiotics was investigated at a uniform 10 μg/ml concentration for all the agents. Experiments were performed in triplicates; mean ± SD are shown, the asterisk (^∗^) above the error bar represents statistically significant differences between the action of the biogenic NPs and the control group (*p*-value < 0.05).

To study the antibacterial efficacy, the biosynthesized AgNPs were tested against six clinically significant pathogenic bacteria, namely *Klebsiella pneumoniae, Pseudomonas brassicacearum, Aeromonas hydrophila, Escherichia coli, Bacillus cereus*, and *Staphylococcus aureus*. Fifty microliters of different concentrations of AgNPs were incubated with the bacterial culture and the zone of inhibition was observed for each case (**Figures 5A–C**). As evidenced from **Figures 5A–C**, the biogenic nanoparticles display consistent antibacterial action against all the types of bacterial pathogens under consideration—even at a very low concentration of 5 μg/ml. Expectedly, the size of inhibition zone increases with the increase in AgNPs concentration as verified through morphometric analysis of the cultures (**Figure 5B**). The antibacterial activity of AgNPs was comparable to the commercial antibiotics at the same concentration (**Figure 5C**), albeit with variations in strength of action of both the AgNP and the antibiotics across the bacterial spectrum.

When AgNPs was mixed with above antibiotics in the same concentration (ca. 10 μg/ml), a substantial increase in the antibacterial activity was observed. To precisely elucidate the nature of synergistic/antagonistic interactions, we computed the interaction index for each combination from the zone of inhibition studies. From **Table 1**, we observe that the order of the degrees of synergistic interaction between these six species was as follows: Pb > Ah > Ec > Bc > Sa > Kp. The highest degree of synergy was found with the three Gram-negative species, Pb, Ah, and Ec, especially for antibiotics rifampicin (R) and streptomycin (S). Little synergy was observed for Kp, even though it is also a Gram-negative species.

**Table 1:** Interaction indices for combination of AgNPs and antibiotics revealing the presence/absence of synergy in inhibiting bacterial growth.

Antibiotics	Interaction Index					
	*Klebsiella pneumoniae* (Kp)	*Pseudomonas brassicacearum* (Pb)	*Aeromonas hydrophila* (Ah)	*Escherichia coli* (Ec)	*Bacillus cereus* (Bc)	*Staphylococcus aureus* (Sa)
Streptomycin (S)	0.95 ± 0.02	2.00 ± 0.12	1.15 ± 0.05	1.17 ± 0.06	1.10 ± 0.06	1.04 ± 0.02
Tetracycline (T)	1.08 ± 0.03	1.26 ± 0.05	1.10 ± 0.02	1.13 ± 0.05	1.19 ± 0.06	1.17 ± 0.06
Kanamycin (K)	0.98 ± 0.02	1.03 ± 0.06	1.22 ± 0.06	1.14 ± 0.05	1.15 ± 0.04	1.07 ± 0.03
Rifampicin (R)	0.98 ± 0.05	1.25 ± 0.06	1.54 ± 0.12	1.54 ± 0.09	1.33 ± 0.09	1.35 ± 0.06

**FIGURE 6:**
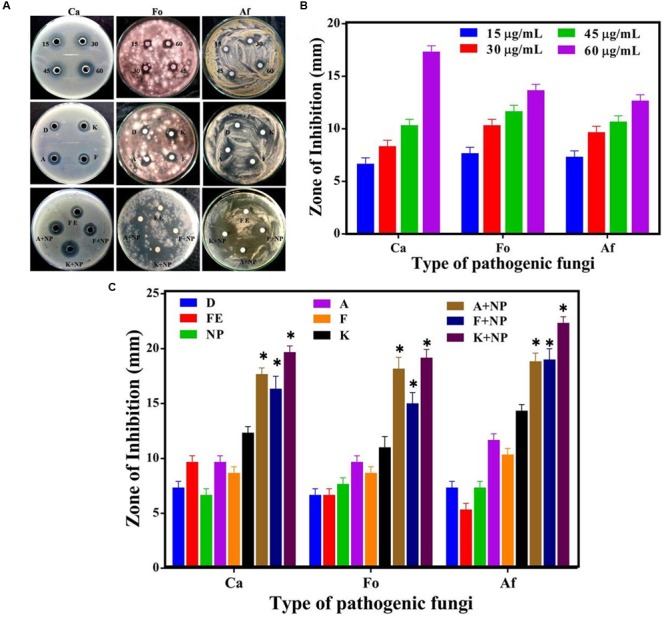
**Comparative analysis of antifungal efficacy of *M. hiemalis*-derived AgNP. (A)** Zone of inhibition measured against three fungal pathogens: *Candida albicans* (Ca), *Fusarium oxysporum* (Fo), *Aspergillus flavus* (Af); **(B)** Measurements of the antifungal activity of AgNPs (NP) at 15, 30, 45, and 60 μg/ml concentration against the three pathogens; and **(C)** Effect of biologically synthesized AgNPs and fungicides [amphotericin B (A), fluconazole (F), and ketoconazole (K)] used singly and in combination against the pathogenic fungi. All measurements were performed for a constant AgNP concentration of 30 μg/ml for commercial fungicides. DMSO (dimethyl sulfoxide) (D) and fungal extract (FE) were employed as negative controls. Experiments were performed in triplicates; mean ± SD are shown, the asterisk (^∗^) above the error bar represents statistically significant differences between the action of the biogenic NPs and the control group (*p*-value < 0.05).

The antifungal characteristic of the synthesized AgNP with three representative fungi, namely *Candida albicans* (Ca), *Fusarium oxysporum* (Fo), and *Aspergillus flavus* (Af) was similarly tested and our observations are shown in **Figure 6**. Morphometric analysis of the incubated cultures exhibits the pattern of antifungal action with increasing concentration of the nanoparticles (**Figure 6B**). The antifungal activity of AgNPs was found to produce similar, if slightly smaller zones of inhibition on average, in relation to that obtained with standard fungicides, namely amphotericin B (A), fluconazole (F), and ketoconazole (K), at the same concentration of 30 μg/ml (**Figure 6C**). Furthermore, when AgNPs was mixed with commercial fungicides, a synergistic antifungal effect was reflected for some combinations. The order of the average degrees of positive interaction between these three species was as follows: Ca > Af > Fo (**Table 2**).

**Table 2:** Interaction indices for combination of AgNPs and fungicides revealing the presence/absence of synergy in inhibiting fungal growth.

Fungicides	Interaction Index		
	*Candida albicans* (Ca)	*Fusarium oxysporum* (Fo)	*Aspergillus flavus* (Af)
Amphotericin B (A)	1.08 ± 0.05	1.04 ± 0.03	0.98 ± 0.05
Fluconazole (F)	1.07 ± 0.03	0.91 ± 0.06	1.08 ± 0.12
Ketoconazole (K)	1.04 ± 0.03	1.03 ± 0.09	1.04 ± 0.03

## Discussion

*Mucor hiemalis*, a common soil fungus of phylum zygomycota, is well known for producing a diverse array of enzymes and lipids (Wang, 1967; Leck, 1999). Based on its rapid growth and ability to withstand a wide spectrum of environmental conditions, we reasoned that *M. hiemalis* can be leveraged as a microbial platform for AgNP synthesis by seizing the Ag^+^ ions from the AgNO_3_ environment and reducing the ions into their elemental form with the help of the enzymes generated through cellular activities supported by Prasad et al. (2016). Extracellular secretion of enzymes is especially advantageous for large-scale nanoparticle synthesis and isolation of nanoparticles produced. The formation and size of AgNPs were also elucidated by observing the surface plasmon resonance peak. The increase in reaction time of AgNPs synthesis results in corresponding blue shifts of the absorption peak, which reflects decrease in the size of the nanoparticles with reaction time, the same condition was also stated by Pandey et al. (2012). Characterization through SEM with EDX revealed consistency in elemental composition of the synthesized nanoparticle. TEM and XRD revealed that the nanoparticles were spherical in shape, crystalline, and well-controlled. It is worth noting that the possibility of aggregate formation of AgNPs, akin to other metal nanoparticles, may lead to functional modifications. The latter can be investigated by quantifying colony formation after incubation in presence of AgNPs in liquid culture and forms an important feature of our ongoing efforts to better understand their antimicrobial properties.

A salient feature of, and often the driving rationale for, a fungal chassis is the intrinsic faster biosynthesis rate in cell-free filtrate as fungi secrete large quantities of extracellular redox proteins capable of reducing metal ions to their insoluble form and subsequently to nanocrystals. It is well established that the high level of enzymes and proteins secreted not only improve the yield but also impart greater stability to the biogenic nanoparticle (Rai et al., 2009). Here, our FTIR findings show the presence of such stabilizing agents and also help identify the biomolecules responsible for Ag^+^ ions reduction to Ag^0^. This finding is not unexpected, as literature reports have consistently observed proteins capping and stabilizing the surface of biogenic nanoparticle (Ahmad et al., 2003; Rai et al., 2009).

### Antimicrobial Activity

The antimicrobial properties of silver are well established and, thus, it has been extensively utilized in the medical field (Wright et al., 1999; Fu et al., 2006). In this study, we first sought to test the relative efficacy of the biogenic nanoparticles, derived from *M. hiemalis*, against a wide spectrum of bacteria and fungi, with respect to the action of commercial antibiotics and fungicides. To study the antibacterial efficacy, the biosynthesized AgNPs were tested against six clinically significant pathogenic bacteria. This cohort was selected to present a challenging and demanding subsets of Gram-positive and Gram-negative bacteria for testing antimicrobial efficacy. This action has been previously explained through a combination of interference with the respiratory chain at the cytochromes (Bragg and Rainnie, 1974) as well as with components of the bacterial electron transport system (Feng et al., 2000), binding DNA and inhibiting its replication (Modak and Fox, 1973).

The antibacterial activity of AgNPs was comparable to the commercial antibiotics at the same concentration, albeit with variations in strength of action of both the AgNPs and the antibiotics across the bacterial spectrum. Additionally, the relative efficacy of the singly used AgNPs was more prominent in the Gram-negative pathogens. Studies by Rhim et al. (2006) and Huang et al. (2011) have spawned similar observations, presumably owing to the thinner cell walls of the Gram-negative strains leading to facile perforation and more rapid absorption. When AgNPs was mixed with above antibiotics in the same concentration (ca. 10 μg/ml), a substantial increase in the antibacterial activity was observed. To precisely elucidate the nature of synergistic/antagonistic interactions, we computed the interaction index for each combination from the zone of inhibition studies. The degree of synergy was lower, on balance, with Gram-positive pathogens reflective of the absence of outer membrane permeability barriers in such bacteria. Collectively, this highlights the presence of synergistic interactions for a large fraction of the AgNPs and antibiotic combinations (rather than simply additive effects) opening the door for combined action against multi-drug resistant strains. This is particularly important in view of the increasing epidemic of drug-resistant pathogens, as several bacteria have developed resistance against conventional antibiotics. It is also notable that biologically synthesized AgNPs have previously shown better synergistic effects as compared to chemically derived AgNPs (Hari et al., 2014). This is attributable to the differential capping of biogenic and chemically synthesized nanoparticles, and is consistent with reports of the direct impact of silver nanomaterial capping on antibiotic sensitive and resistant strains of bacteria (Tiwari et al., 2015).

One plausible explanation for the enhanced antibacterial activity using a combination of AgNP and antibiotics is the bonding between the nanoparticles and antibiotic molecules. The active functional groups of antibiotics such as hydroxyl and amino groups, can be chelated by silver and thereby cover a considerable portion of the surface of AgNPs (Morones-Ramirez et al., 2013). Alternately, the findings can also be driven by the AgNP-induced reactive oxygen species generation that increases the cell permeability, which in turn stimulates the antibiotic action (Fayaz et al., 2010).

In relation to the antifungal activity, the degree of synergy was lowest with *Fusarium oxysporum*, reflecting the characteristic of the constituents present on the surface of the fungi causing permeability barriers. While the synergy in most combinations was found to be statistically significant, it is worth noting that the degree of synergy was not as strong as that noted in some of the antibacterial studies.

## Conclusion

In the quest for advanced topical ointments, wound healing bandages and coated stents with superior resistance to microbial infections, nanosilver formulations have surfaced as an attractive option. With an increasing focus on less toxic and cleaner synthesis methods that require minimal downstream processing, we report here a fungal platform, *M. hiemalis*, which facilitates rapid and large-scale production of well-controlled AgNPs. Characterization of these biogenic NPs highlights not only their crystalline nature but also reveals the presence of capping and stabilizing proteins on the surface thereby enabling its direct use for antimicrobial coatings. In our study, we also observed that these NPs show competitive inhibitory effect on a broad spectrum of pathogenic bacteria and fungi in relation to antibiotics and fungicides, which cannot (individually) mimic the action against both set of pathogens. Furthermore, the AgNPs combined with the antibiotics and fungicides have a significant synergistic effect on these species. Overall, these findings underscore the promise of the *M. hiemalis* derived AgNPs, and provide the scientific foundation for translational studies in animal models.

## Author Contributions

RaP and IB conceived and designed the experiments; NA performed the experiments; RaP, RiP, and IB analyzed the data; NA prepared the draft; and RaP, RiP, and IB proofread the final draft. All authors approved the final manuscript.

## Conflict of Interest Statement

The authors declare that the research was conducted in the absence of any commercial or financial relationships that could be construed as a potential conflict of interest.
