# Supplementary material for: Leveraging the Attributes of Mucor hiemalis-Derived Silver Nanoparticles for a Synergistic Broad-Spectrum Antimicrobial Platform
Source: Front Microbiol. 2016 Dec 15;7:1984. doi: 10.3389/fmicb.2016.01984 (PMC5156874; doi:10.3389/fmicb.2016.01984)
Supplement: Supplementary file 1 [file Table_1.DOC]

**Table S1.** Percentage by weight of different elements in the biosynthesized silver nanoparticles, as revealed by EDX analysis.

| **Element** | **Weight%** | **Atomic%** |
| --- | --- | --- |
| **C** | 0.96 | 5.88 |
| **O** | 6.09 | 27.82 |
| **Na** | 1.31 | 4.15 |
| **Ag** | 91.64 | 62.15 |
| **Total** | 100 |  |
